# Editorial: Plant stress resistance: unraveling the mechanisms and strategies for resilience

**DOI:** 10.3389/fpls.2026.1817950

**Published:** 2026-03-13

**Authors:** Yu-Tao Bao, Kai-Hua Jia, Ren-Gang Zhang, Wei Zhao, Longxin Wang

**Affiliations:** 1 School of Biological Science and Technology, University of Jinan, Jinan, China; 2 College of Biological Sciences and Technology, Beijing Forestry University, Beijing, China; 3 Institute of Crop Germplasm Resources, Shandong Academy of Agricultural Sciences, Jinan, China; 4 Yunnan Key Laboratory for Integrative Conservation of Plant Species with Extremely Small Populations/Key Laboratory for Plant Diversity and Biogeography of East Asia, Kunming Institute of Botany, Chinese Academy of Sciences, Kunming, China; 5 Laboratory of Tropical Forest Ecology, Xishuangbanna Tropical Botanical Garden, Chinese Academy of Sciences, Xishuangbanna, China

**Keywords:** abiotic stress, biotechnology, crop resilience, molecular mechanisms, stress resistance

## Introduction

Driven by the exacerbation of global climate change, plants are increasingly subjected to severe and unpredictable abiotic stresses, such as drought, extreme temperatures, soil salinization, and heavy metal toxicity. At the same time, the pressure to sustain a growing global population necessitates agricultural systems that are both highly productive and environmentally resilient. Understanding how plants perceive stress signals, reprogram their molecular networks, and enact physiological and anatomical adaptations is crucial. This Research Topic, “Plant Stress Resistance: Unraveling the Mechanisms and Strategies for Resilience”, brings together 20 significant contributions, comprising 17 original research articles and 3 extensive reviews, which span from foundational molecular and multi-omics discoveries to innovative biotechnological and agronomic applications.

## Decoding the genomic and molecular architecture of stress tolerance

Breeding climate-resilient crops requires a clear understanding of their genetic blueprints and regulatory networks that govern stress responses. Several studies in this Research Topic utilized genome-wide approaches and transcriptomics to identify critical resilience hubs. For instance, Parra et al. assembled a high-quality haploid reference genome for Hayward kiwi and identified *AdhSAP4*, a stress-associated protein that acts as a negative regulator of salinity tolerance. Complementing these findings, systematic genome-wide characterizations elucidated the evolutionary and functional dynamics of the TCP transcription factor family in pea (Song et al.) and the LBD gene family in sour jujube Li et al., pinpointing specific hub genes, such as *PsTCP20*, that regulate photosynthesis and metabolic adjustments under stress.

Exploring adaptations to extreme environments, Jiang et al. conducted comparative transcriptomics on Tibetan qingke (hulless barley), revealing gene modules uniquely tied to photosynthetic preservation and reactive oxygen species (ROS) scavenging under drought. Translating multi-omics into targeted QTL mapping, Liu et al. mapped *qESCT2* in rice and identified *OsWRKY71*, a transcription factor that regulates early-seedling cold tolerance via glutathione metabolism pathways.

Functional validations of specific genes further highlighted the complexity of stress networks. Cao et al. explored heterosis in maize, demonstrating that the non-additive gene *ZmbHLH137* positively regulates drought tolerance by enhancing antioxidant enzyme activities. Similarly, silencing specific *HSP90* genes in cotton strongly impaired ROS scavenging, confirming their vital role in salt stress adaptation Hao et al. In addition, environmental stress often overlaps with biotic threats; Du et al. demonstrated that silencing the *StCCR6* gene in potato not only altered lignin biosynthesis but also exacerbated susceptibility to bacterial infection, underscoring the key crosstalk between abiotic and biotic defense pathways.

## Physiological homeostasis, structural remodeling, and growth trade-offs

To survive environmental perturbations, plants must translate molecular signals into tangible physiological and structural adaptations. A detailed review by Tariq et al. synthesized the integrative dynamics of cell wall architecture under salt stress, emphasizing how anisotropic morphogenesis and altered polysaccharide compositions maintain cell integrity. At the organ level, anatomical evaluations by Soliman et al. revealed that drought-tolerant soybean genotypes thrive under water-deficit conditions by maintaining thicker primary and secondary xylem tissues and preserving stem integrity.

At the cellular and membrane levels, Ramalho et al. provided a deep dive into the transcriptomic, proteomic, and lipidomic responses of *Coffea* species facing superimposed heat and drought. Their findings highlighted *de novo* lipid synthesis and the dynamic remodeling of chloroplast membranes as key survival mechanisms. Xu et al. also demonstrated that overexpressing the *Salix matsudana* aquaporin gene *SmPIP1;3* in tobacco alleviated multi-stress damage by sustaining physiological homeostasis and shielding membranes from oxidative stress.

Notably, stress resilience is not without its costs, often involving complex growth-stress trade-offs. Deng et al. identified that PSY peptides in tobacco promote seed germination and vegetative growth but inadvertently impair osmotic stress tolerance. Adding another layer of complexity, Kobayashi et al. uncoupled the canonical DNA repair function of the *UVH6* gene in *Arabidopsis*, revealing its distinct, novel role in coordinating transcriptional responses to osmotic and heat stress.

## Biochemical defenders: metabolites and exogenous elicitors

The biochemical landscape of plants, particularly the accumulation of specific metabolites, acts as a frontline defense against oxidative damage. A review by Basit et al. extensively documented the complex role of glycine betaine in mitigating stress by detoxifying ROS and stabilizing photosynthetic machinery. Jangpangi et al. reviewed the substantial impact of climate change on medicinal plants, highlighting how extreme temperatures and elevated CO_2_ modulate the synthesis of therapeutically valuable plant secondary metabolites (PSMs), an area critical for both plant adaptation and human pharmacology.

Beyond endogenous synthesis, exogenous application of bioactive molecules offers a practical agronomic strategy. Zhang et al. demonstrated that foliar application of melatonin synergistically enhances drought resistance in cotton. This resilience was anatomically backed by the expansion of the living cortical area (LCA) in roots, which directly correlated with improved water absorption, osmotic regulation, and respiratory metabolism.

## Innovative nanotechnological and ecological interventions

To transition basic science to practical field solutions, this Research Topic highlights cutting-edge interventions. Nanotechnology represents a transformative frontier in agriculture. Shah et al. showed that applying magnesium oxide nanoparticles (MgONPs) significantly alleviated cadmium toxicity in moso bamboo. The MgONPs repaired ultrastructural damage in guard cells and boosted oxidative defense mechanisms. Wang et al. synthesized a licorice-wolfberry-derived complex nanomaterial (LW-CNs) that markedly improved wheat germination under salt stress by maintaining ROS homeostasis and optimizing the internal K+/Na+ ratio.

Ecological approaches also proved highly effective for sustainable forestry and agriculture. Mu et al. pangi leveraged plant-microbe symbiosis by inoculating the short-rotation woody crop *Paulownia elongata* with the endophytic fungus *Piriformospora indica*. This interaction significantly reduced plant mortality under high salinity by enhancing photosynthetic rates and antioxidant enzyme activities.

## Conclusion

The 20 articles compiled in this Research Topic collectively show the remarkable plasticity and resilience of plants. From assembling genomes and mapping crucial QTLs to uncovering the nuances of cell wall remodeling, metabolic shifts, and nanoparticle-assisted phytoremediation, these studies advance our foundational knowledge of stress biology. More importantly, they provide a diverse toolkit, ranging from genetic engineering and marker-assisted breeding to exogenous elicitors and beneficial microbes, for developing next-generation crops. We extend our deepest gratitude to all authors and reviewers for their invaluable contributions. It is our hope that these insights will serve as a foundation for future molecular breeding and climate-smart agricultural practices, ultimately fostering a more resilient and sustainable agricultural future.

